# Myocardial involvement in eosinophilic granulomatosis with polyangiitis evaluated with cardiopulmonary magnetic resonance

**DOI:** 10.1007/s10554-020-02091-1

**Published:** 2020-11-19

**Authors:** Jakub Lagan, Josephine H. Naish, Christien Fortune, Joshua Bradley, David Clark, Robert Niven, Nazia Chaudhuri, Erik B. Schelbert, Matthias Schmitt, Christopher A. Miller

**Affiliations:** 1 Manchester University NHS Foundation Trust, Wythenshawe Hospital, Southmoor Road, Wythenshawe, Manchester M23 9LT UK; 2 Division of Cardiovascular Sciences, School of Medical Sciences, Faculty of Biology, Medicine and Health, Manchester Academic Health Science Centre, University of Manchester, Oxford Road, Manchester, M13 9PL UK; 3 Division of Infection, Immunity and Respiratory Medicine, School of Biological Sciences, Faculty of Biology, Medicine and Health, Manchester Academic Health Science Centre, University of Manchester, Oxford Road, Manchester, M13 9PL UK; 4 Department of Medicine, University of Pittsburgh School of Medicine, Pittsburgh, PA USA; 5 UPMC Cardiovascular Magnetic Resonance Center, Heart and Vascular Institute, Pittsburgh, PA USA; 6 Clinical and Translational Science Institute, University of Pittsburgh, Pittsburgh, PA USA; 7 Division of Cell-Matrix Biology and Regenerative Medicine, School of Biology, Faculty of Biology, Medicine and Health, Manchester Academic Health Science Centre, Wellcome Centre for Cell-Matrix Research, University of Manchester, Oxford Road, Manchester, M13 9PT UK

**Keywords:** Cardiac magnetic resonance scanning, Parametric mapping, Dynamic contrast enhanced magnetic resonance, Eosinophilic granulomatosis with polyangiitis, Churg, Strauss syndrome

## Abstract

Patients with eosinophilic granulomatosis with polyangiitis (EGPA) most commonly die from cardiac causes, however, cardiac involvement remains poorly characterised and the relationship between cardiac and pulmonary disease is not known. This study aimed to characterise myocardial and pulmonary manifestations of EGPA, and their relationship. Prospective comprehensive cardiopulmonary investigation, including a novel combined cardiopulmonary magnetic resonance imaging (MRI) technology, was performed in 13 patients with stable EGPA. Comparison was made with 11 prospectively recruited matched healthy volunteers. Stable EGPA was associated with focal replacement and diffuse interstitial myocardial fibrosis (myocardial extracellular volume 26.9% vs. 24.7%; p = 0.034), which drove a borderline increase in left ventricular mass (56  ±  9 g/m2 vs. 49  ±  8 g/m2; p = 0.065). Corrected QT interval was significantly prolonged and was associated with the severity of myocardial fibrosis (r = 0.582, p = 0.037). Stable EGPA was not associated with increased myocardial capillary permeability or myocardial oedema. Pulmonary tissue perfusion and capillary permeability were normal and there was no evidence of pulmonary tissue oedema or fibrosis. Forced expiratory volume in one second showed a strong inverse relationship with myocardial fibrosis (r = −0.783, p = 0.038). In this exploratory study, stable EGPA was associated with focal replacement and diffuse interstitial myocardial fibrosis, but no evidence of myocardial or pulmonary inflammation or pulmonary fibrosis. Myocardial fibrosis was strongly associated with airway obstruction and abnormal cardiac repolarisation. Further investigation is required to determine the mechanisms underlying the association between heart and lung disease in EGPA and whether an immediate immunosuppressive strategy could prevent myocardial fibrosis formation.

## Introduction

Eosinophilic granulomatosis with polyangiitis (EGPA), formerly known as Churg-Strauss syndrome, is a hybrid condition comprising a systemic antineutrophil cytoplasmic antibody (ANCA)-associated vasculitis and a hypereosinophilic disorder with frequent lung involvement that is associated with asthma [1, 2].

Whilst a wide range of cardiac manifestations are described, including pericarditis, pericardial effusion, tamponade, myocarditis, cardiomyopathy, myocardial infarction and heart failure [2], clinically evident cardiac manifestations are uncommon [3, 4]. In contrast, up to approximately 90% of patients have evidence of cardiac involvement at post-mortem and between a third and three quarters of patients with EGPA die from cardiac causes [4–7].

Magnetic resonance imaging (MRI) provides unparalleled evaluation of cardiac structure, function and tissue characterisation and its application in EGPA has provided insight into cardiac disease expression [5, 8–11]. However, contemporary cardiac MRI techniques, which provide quantitative characterisation of myocardial injury and adaptation, have been applied sparsely and the relationship between cardiac and pulmonary disease is not known.

In this study, we sought to apply a novel combined cardiopulmonary MRI technology, which provides comprehensive and simultaneous interrogation of cardiac and pulmonary structure, function and tissue character, in patients with stable EGPA in order to: (1) Characterise myocardial and pulmonary manifestations of EGPA; and (2) Investigate the relationship between cardiac and pulmonary disease in EGPA.

## Materials and methods

### Study design

This prospective research study aimed to apply a combined cardiopulmonary MRI protocol in patients with stable EGPA and matched healthy volunteers in order to investigate myocardial and pulmonary manifestations of EGPA, and their relationship.

### Study population

Stable patients with a clinical diagnosis of EGPA according to the 2012 Chapel Hill consensus [1] were prospectively recruited at Manchester University NHS Foundation Trust. Age and gender matched volunteers with no cardiovascular symptoms, no history of any medical condition and normal electrocardiogram (ECG) were prospectively recruited as controls.

Exclusion criteria included: history of any cardiovascular disorder other than cardiac disease that was thought, by the attending clinical team, to be secondary to EGPA, contraindication to MRI, hypersensitivity to gadolinium-based contrast agent (GBCA), estimated glomerular filtration rate < 40 ml/min/1.73m^2^.

### Study procedures

Participants underwent blood sampling for blood count, renal function, c-reactive protein, high sensitivity troponin I, and B-human chorionic gonadotropin in females of childbearing potential, ECG and MRI scanning on the same day (see below). Clinical pulmonary function tests performed closest to the study visit were recorded.

### Combined cardiopulmonary MRI

#### Protocol

**Fig. 1:**
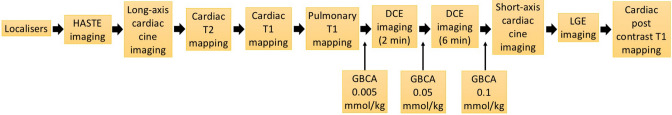
Cardiopulmonary magnetic resonance imaging protocol. *DCE* dynamic contrast enhanced, *GBCA* gadolinium based contrast agent, *HASTE* half-fourier-acquired single-shot turbo spin echo, *LGE* late enhancement imaging

MRI was performed at 1.5T (Avanto, Siemens Medical Imaging). Scan duration 60 min. It included the following sequences (Fig. 1):

1. Steady-state free precession (SSFP) cine imaging of the heart (standard long- and short-axis views) to provide measurement of cardiac function.
2. T1 mapping (by MOdified Look-Locker Inversion Recovery (MOLLI)) at basal and mid left ventricular (LV) short axis level before and 15 min following the final bolus of GBCA (see below) to provide evaluation of myocardial oedema and fibrosis. T2 mapping (by T2-prepared SFFP) at the same locations to provide further assessment of myocardial oedema. Late gadolinium enhancement (LGE) imaging beginning at 6 min following the final GBCA bolus (see below) to assess for focal replacement myocardial fibrosis.
3. T1 mapping (by MOLLI) in the central sagittal plane of both lungs to provide evaluation of lung tissue injury.
4. Dynamic contrast enhanced (DCE) imaging using free-breathing 2D saturation-recovery fast low angle shot (FLASH) dynamic acquisitions in four planes (basal and mid LV short axis, left and right lung central sagittal planes) to assess myocardial and pulmonary capillary permeability (transfer constant (K^trans^)), pulmonary extracellular volume fraction (Ve) and blood flow (F). GBCA (gadoterate meglumine (Dotarem), Guerbet, France; 3 ml/s) administration was divided into three doses in order to avoid saturation of the peak of the arterial input function (AIF), which occurs as a result of the non-linear relationship between contrast agent concentration and signal intensity, and T2 shortening effects, at higher contrast agent concentrations [12]. The initial low dose (0.005 mmol/kg) bolus was used to calculate the AIF first pass peak, followed by 2 min of dynamic acquisitions with a temporal resolution equal to 50% of heart rate. Then a higher GBCA dose (0.05 mmol/kg) was administered and followed by 6 min of dynamic acquisitions. The higher dose provided higher contrast to noise ratio for the tail of the AIF and for the myocardial and pulmonary tissue curves. The low dose curve was multiplied by 10 and combined with the high dose curve to produce the final AIFs for the kinetic analysis. The third contrast agent dose (0.1 mmol/kg) was administered after the dynamic acquisition to allow LGE imaging and post-contrast T1 mapping.

#### Analysis

##### Myocardial function

Cardiac volumetric analysis was performed using Circle CVI42 (Circle Cardiovascular Imaging, Canada) according to current guidelines [13].

##### Myocardial and lung tissue characterisation

T1 and T2 maps were generated in Siemens Argus (Siemens Medical Imaging) and transferred into Horos (Horos2K v2.2.0 The Horos Project) where epicardial, endocardial and blood pool regions of interest (ROI) were drawn. Partial volume effects of blood were minimised in myocardial ROIs by using the middle third of myocardium. Lung borders were contoured in Horos, excluding the cardiac outline, to define whole-lung ROI. Individual lobes were identified as previously described [14]. Myocardial extracellular volume (ECV) was calculated using same-day haematocrit as described previously [15]. LV extracellular matrix mass (g) was calculated by multiplying LV mass by ECV [16]. LV cellular mass (g) was calculated by multiplying LV mass by (100%-ECV).

##### Myocardial and lung blood flow characteristics

DCE imaging was analysed using custom written Matlab code (v9.0, The MathWorks, USA). Cardiac motion correction was achieved using an intensity-based rigid registration algorithm. Lung registration was performed using Advanced Normalisation Tools (ANTs) symmetric normalization non-linear registration algorithm, employing cross-correlation as the similarity measure [17]. Epicardial, endocardial and lung ROIs were drawn as described above. AIFs were derived from the right ventricular (RV) blood pool for the lungs and the LV blood pool for the myocardium. Care was taken to avoid any trabeculations or papillary muscles. Contrast agent kinetics were modelled using an extended version of a Kety model on a voxel-by-voxel basis within registered ROIs to calculate K^trans^ and Ve [18]. Pulmonary blood flow (F) was calculated by deconvolution of the first pass dynamic data as described previously [18, 19].

### Statistical analysis

This was an exploratory study, and there were limited data upon which to base a power calculation. As a guide, 10 patients were required in each group to detect an absolute minimum difference, between EGPA and healthy control groups, in myocardial ECV of 3% [10], with 80% power at a 5% significance level (2-sided), assuming a standard deviation of 2.2% within each group [10, 11, 15]. Data distribution was determined using the Shapiro–Wilk test. Data are summarised using mean and standard deviation or median and interquartile range (IQR), and were compared using t tests or non-parametric equivalents, as appropriate. Analyses were performed using SPSS (version 22, IBM, USA).

## Results

### Participant characteristics

Thirteen patients with stable EGPA and 11 healthy volunteers were recruited (Table 1). Groups were matched for age and gender. Twelve (92%) patients with EGPA were receiving immunosuppressive therapy at the time of the study. Patients with EGPA had a significantly higher white cell count (10.7 vs. 6.0 × 10^9^/L; p = 0.001), however, eosinophil count was no different (0.23 vs. 0.11 × 10^9^/L; p = 0.23). High sensitivity troponin I was within the normal range in all participants. Pulmonary function tests were available in seven patients with EGPA.

**Table 1:** Participant characteristics

Parameter	Controls (n = 11)	EGPA (n = 13)	p value
Demographics			
Age (years)	53 (46–56)	54 (49–57)	0.543
Gender (female, %)	4 (36%)	6 (46%)	0.697
BSA (m^2^)	1.98 (1.73–2.21)	2.22 (1.8–2.3)	0.369
EGPA manifestations			
ANCA positive	0 (0%)	3 (23%)	
Asthma	0 (0%)	12 (92%)	
History of eosinophilia	0 (0%)	13 (100%)	
Peripheral neuropathy	0 (0%)	10 (77%)	
Paranasal sinus abnormalities	0 (0%)	6 (46%)	
Extravascular eosinophils	0 (0%)	2 (15%)	
Cardiac involvement	0 (0%)	2 (15%)	
Renal involvement	0 (0%)	2 (15%)	
GI involvement	0 (0%)	2 (15%)	
Dermatological involvement	0 (0%)	2 (15%)	
Polymyalgia	0 (0%)	2 (15%)	
Hypertension	0 (0%)	2 (15%)	
Alveolar haemorrhage	0 (0%)	1 (8%)	
Medications			
Oral steroids	0 (0%)	12 (92%)	
Mycophenolate mofetil	0 (0%)	6 (46%)	
Methotrexate	0 (0%)	2 (15%)	
Inhaled steroids	0 (0%)	11 (85%)	
Inhaled LABAs	0 (0%)	9 (69%)	
Inhaled SABAs	0 (0%)	12 (92%)	
Inhaled LAMAs	0 (0%)	4 (31%)	
Theophylline	0 (0%)	2 (15%)	
LTRA	0 (0%)	2 (15%)	
Macrolides	0 (0%)	3 (23%)	
Statins	0 (0%)	4 (31%)	
ACEI/sartans	0 (0%)	3 (23%)	
B Blockers	0 (0%)	1 (8%)	
Calcium channel blockers	0 (0%)	1 (8%)	
Duretics	0 (0%)	3 (23%)	
Antiplatelets	0 (0%)	1 (8%)	
Anticoagulants	0 (0%)	2 (15%)	
Lung function tests			
FEV_1_ (L/s)		2.4 ± 0.7*	
FEV_1_% (%)		70.6 ± 25.0*	
FVC (L)		3.6 ± 1.0*	
FVC% (%)		85.0 ± 18.8*	
FEV_1_/FVC		0.68 ± 0.13*	
Laboratory findings			
CRP (mg/L)	1 (0–2)	3 (1–10)	0.070
WBC (× 10^9^/L)	6 (4.9–7.1)	10.7 (9.8–12.85)	0.001
Eosinophil count (× 10^9^/L)	0.11 (0.07–0.21)	0.23 (0.07–0.44)	0.213
Troponin I (ng/L)	4 (4–4)	4 (4–4)	0.317
ECG findings			
PR duration (ms)	160 (130–160)	160 (140–160)	0.975
QRS duration (ms)	80 (80–100)	80 (70–80)	0.115
QTc (ms)	398 ± 18	414 ± 17	0.043
ST-T abnormalities (present)	0 (0%)	4 (31%)	0.098

Data presented as mean ± standard deviation or median (interquartile range) depending on data distribution

*ACEI* angiotensin converting enzyme inhibitor, *ANCA* antineutrophil cytoplasmic antibodies, *BSA* body surface area, *CRP* c reactive protein, *EGPA* eosinophilic granulomatosis with polyangiitis, *FEV*_*1*_ forced expiratory volume in one second, *FEV*_*1*_% percentage of predicted forced expiratory volume in one second, *FVC* forced vital capacity, *FVC%* percentage of predicted forced vital capacity GI-gastrointestinal tract, *LABA* long acting B agonist, *LAMA* long acting muscarinic antagonist, *LTRA* leukotriene receptor antagonist, *LVH* left ventricular hypertrophy, *SABA* short acting B agonist, *WBC* white blood cell

^*^N = 7

Two patients with EGPA had a history of cardiac involvement; one patient had previously had a pericardial effusion and a pericardial window formed, one patient had experienced a distal coronary artery dissection at a young age.

Corrected QT interval was significantly longer in EGPA than in healthy volunteers (414  ±  17 vs. 398  ±  18 ms; p = 0.04). Four patients with EGPA exhibited ST segment (ST elevation in one; ST depression in one) or T wave abnormalities (T wave inversion in two). No patients exhibited clinically significant arrhythmias.

### Cardiopulmonary manifestations

Ventricular size, ventricular ejection fraction and atrial size were no different in EGPA compared to control (Table 2). Body surface area-indexed LV mass was borderline higher in EGPA (56  ±  9 vs. 49  ±  8 g/m2; p = 0.065).

**Table 2:** Myocardial magnetic resonance imaging indices

Parameter	Controls (n = 11)	EGPA (n = 13)	p value
LV			
LV EDV/BSA (ml/m^2^)	76 ± 11	76 ± 18	0.932
LV ESV/BSA (ml/m^2^)	30 ± 6	31 ± 14	0.770
LV EF (%)	62 ± 5	60 ± 9	0.645
LV mass/BSA (g/m^2^)	49 ± 8	56 ± 9	0.065
RV			
RV EDV/BSA (ml/m^2^)	83 ± 14	77 ± 16	0.305
RV ESV/BSA (ml/m^2^)	37 ± 7	33 ± 10	0.396
RV EF (%)	56 ± 4	56 ± 8	0.996
Pericardium			
Pericardial effusion (n,%)	0 (0%)	1 (8%)	1.000
Pericardial thickening (n,%)	0 (0%)	2 (15%)	0.482
Atria			
LA area/BSA (cm^2^/m^2^)	12 ± 1	12 ± 3	0.319
RA area/BSA (cm^2^/m^2^)	12 ± 3	11 ± 2	0.221
Tissue characterisation			
Ischaemic LGE (present)	0 (0%)	2 (15%)	0.482
Ischaemic LGE (g)	0.00 (0.00–0.00)	0.00 (0.00–0.00)	0.184
Non-ischaemic LGE (present)	0 (0%)	10 (77%)	< 0.001
Non-ischaemic LGE (g)	0.00 (0.00–0.00)	0.51 (0.12–3.16)	0.001
T2 (ms)	50 ± 2	50 ± 3	0.533
T1 (ms)	1003 ± 21	1063 ± 46	0.001
ECV (%)	24.7 (23.6–26.4)	26.9 (25.9–28.5)	0.034
Extracellular matrix mass (g)	24 ± 6	32 ± 9	0.015
Cellular mass (g)	74 ± 21	84 ± 16	0.191
Median K^trans^ (min^−1^)	0.32 ± 0.08	0.31 ± 0.08	0.762

Data presented as mean ± standard deviation or median (interquartile range) depending on data distribution

*ECV* extracellular volume fraction, *EDV* end systolic volume, *EF* ejection fraction, *ESV* end systolic volume, *K*^*trans*^ transfer constant, *LA* left atrium, *LGE* late gadolinium enhancement, *LV* left ventricle, *RA* right atrium, *RV* right ventricle, *SV* stroke volume. Other abbreviations as per Table 1

**Fig. 2:**
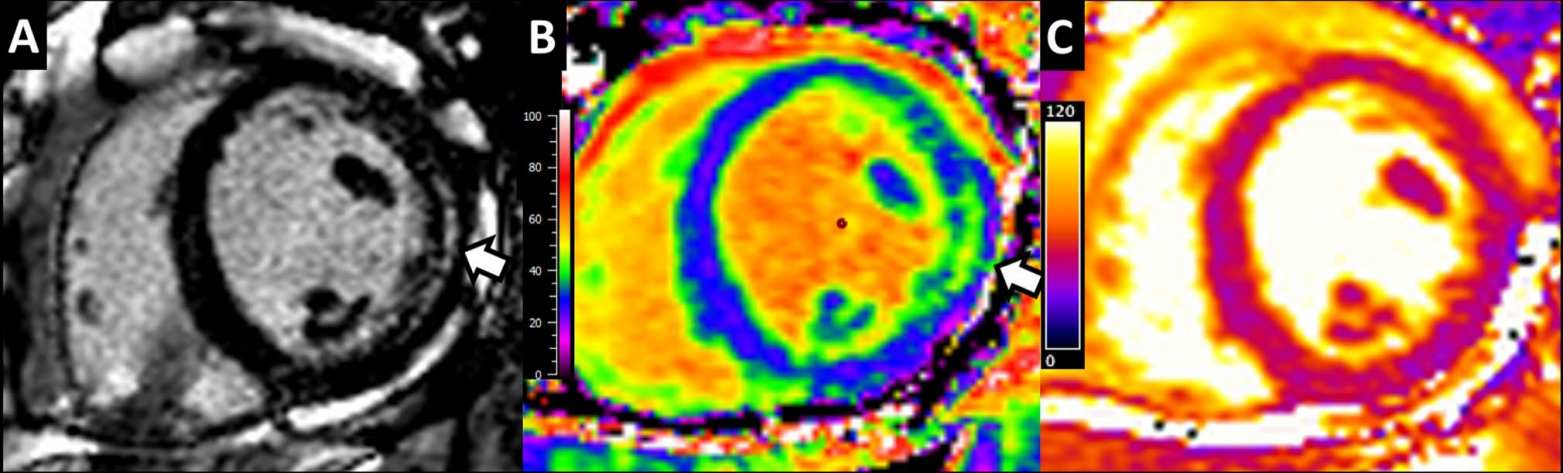
Myocardial tissue characterisation in a patient with eosinophilic granulomatosis with polyangiitis. **a** On late enhancement imaging (arrow) and **b** extracellular volume mapping (extracellular volume in the lateral wall is elevated at 37%), there is evidence of non-ischaemic fibrosis in the lateral wall of the left ventricle. **c** On T2 mapping, there is no evidence of myocardial inflammation (lateral wall: 45 ms; septum: 47 ms)

EGPA was associated with elevated myocardial ECV (26.9% (25.9–28.5%) vs 24.7% (23.6–26.4%); p = 0.034), increased extracellular matrix mass (24  ±  6 vs. 32  ±  9; p = 0.015) and elevated native T1 (1063 ± 46 vs. 1003  ±  21 ms; p = 0.001). Ten patients with EGPA exhibited non-ischaemic LGE (mid myocardial wall in five, epicardial in one, RV insertion point in nine; p < 0.001; (some patients displayed more than one type)). Myocardial T2 and capillary permeability were no different in EGPA compared to control. Figure 2 illustrates representative examples of tissue characterisation in a patient with EGPA.

**Fig. 3:**
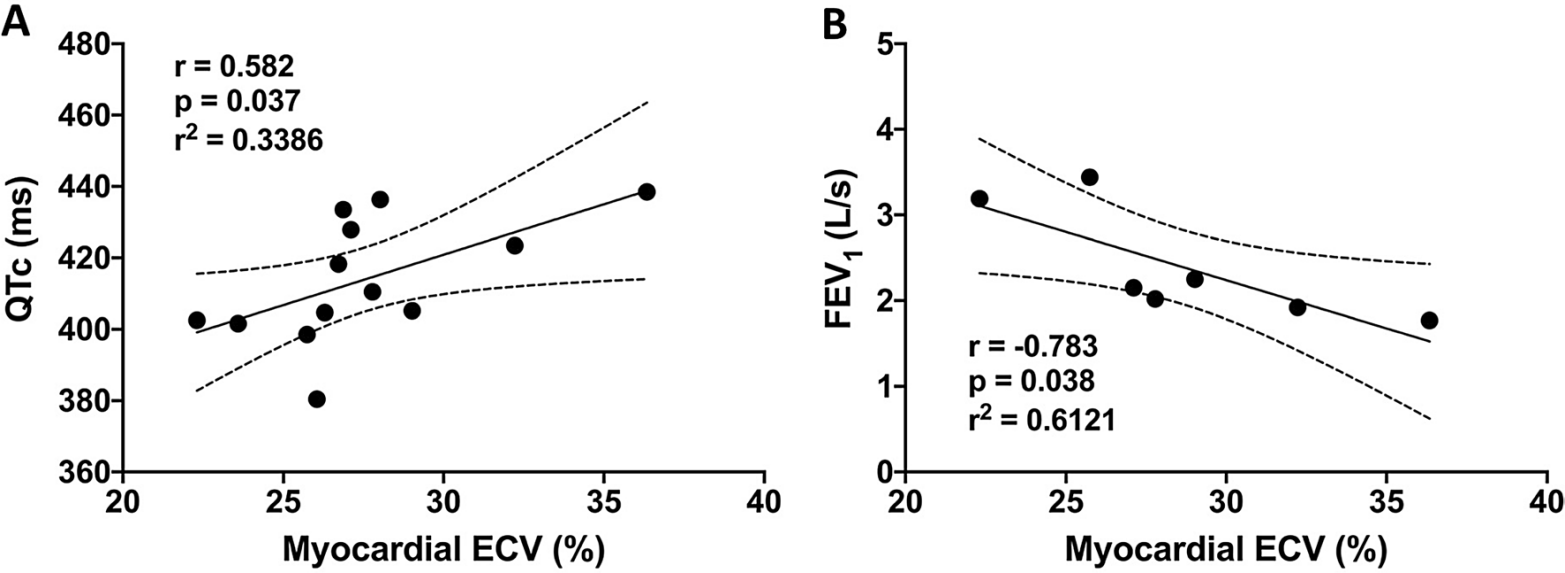
Relationship between myocardial extracellular volume (ECV) fraction, lung function and cardiac repolarisation in eosinophilic granulomatosis with polyangiitis. **a** Corrected QT interval was significantly associated with myocardial fibrosis burden, measured using extracellular volume fraction (ECV). **b** Forced expiratory volume in one second (FEV_1_) was significantly negatively correlated to myocardial fibrosis burden, measured using ECV

There was no relationship between corrected QT interval and medications. Corrected QT interval was significantly associated with myocardial ECV interval (r = 0.582, p = 0.037; Fig. 3a).

Two patients had evidence of a myocardial infarction (MI) on LGE imaging: in one patient it was previously unrecognised and the other was the patient with a history of coronary artery dissection.

Pulmonary tissue T1, capillary permeability, extracellular volume fraction and blood flow were no different in EGPA compared to healthy volunteers (Table 3).

**Table 3:** Pulmonary magnetic resonance imaging indices

Parameter	Controls (n = 11)	EGPA (n = 13)	p value
T1 (ms)	1261 ± 46	1290 ± 65	0.232
Median Ve (%)	20.8 (18.73–26.86)	21.9 (18.62–25.64)	0.839
Median K^trans^ (min^−1^)	0.25 ± 0.07	0.23 ± 0.09	0.561
Median F (ml blood/ml tissue/min)	2.32 (1.76–3.10)	2.77 (1.88–3.15)	0.794

Data presented as mean ± standard deviation or median (interquartile range) depending on data distribution

*F* Pulmonary tissue blood flow, *Ve* pulmonary extracellular volume fraction. Other abbreviations as per Table 2

### Relationship between cardiac and pulmonary manifestations

Forced expiratory volume in one second (FEV_1_) was strongly negatively correlated to native myocardial T1 (r = −0.816, p = 0.025) and ECV (r = −0.783, p = 0.038; Fig. 3b). There were no significant correlations between myocardial and pulmonary MRI measurements, and no association between circulating eosinophil levels and cardiac findings.

## Discussion

In the first study to utilise a combined multiparametric cardiopulmonary MRI protocol in EGPA, we demonstrate a number of novel findings. We show that stable EGPA is associated with focal replacement and diffuse interstitial myocardial fibrosis, which drives a borderline increase in LV mass, but is not associated with myocardial oedema or increased capillary permeability. We show that myocardial fibrosis burden is associated with abnormal cardiac repolarisation. We demonstrate that in the setting of stable disease, EGPA is associated with normal pulmonary tissue perfusion and no evidence of pulmonary inflammation or fibrosis. Finally, we show that myocardial fibrosis burden is strongly related to the severity of airway obstruction.

It is increasingly recognised that conditions considered to be primary pulmonary disorders are associated with cardiovascular conditions, particularly heart failure, independent of shared risk factors, and that in turn, cardiovascular conditions independently contribute to all-cause mortality in patients with pulmonary conditions [20].

EGPA has dual disease categorisation, being classified within the small to medium vessel vasculitides associated with ANCA and in the hypereosinophilic syndromes [2]. It is characterised histologically by the presence of a small to medium vessel necrotising vasculitis and eosinophil-rich, necrotising granuloma, which often involve the respiratory tract. Both vessel inflammation and eosinophilic proliferation are thought to contribute to organ damage [1].

The cardiac pathogenesis is that of eosinophilic myocardial infiltration that rapidly progresses to myocardial fibrosis, which chronically leads to dilated or restrictive cardiomyopathy [2]. Cardiac involvement often occurs early in the disease process [8]. Some studies report that cardiac involvement is more common in ANCA-negative patients and is related to the degree of blood eosinophilia, but others have not found these associations [4, 8, 21]. Cardiomyopathy is independently associated with early death in patients with EGPA, and cardiac involvement potentially accounts for up to 80% of deaths [2, 3, 7].

Patients all had stable EGPA, as evidenced by low levels of circulating C reactive protein and eosinophil counts (the slightly elevated white cell count is likely because 92% of patients were receiving corticosteroids). Perhaps as a result, we did not find an association between cardiac manifestations and circulating eosinophil levels. Seventy-seven percent of patients were ANCA-negative at diagnosis.

The multiparametric nature of MRI, i.e. its ability to measure multiple aspects of tissue structure, function, injury and adaptation within one scan, without radiation, means that it is uniquely placed to elucidate underlying disease mechanisms and understand their relationships. Myocardial ECV, measured using cardiac MRI, quantifies myocardial extracellular matrix volume and in the absence of significant inflammation, is a robust measure of myocardial fibrosis, having been extensively validated against histological collagen volume fraction [16, 22, 23]. Myocardial T1 is a surrogate of ECV, having also been validated against histological collagen. Myocardial LGE provides robust identification of focal replacement fibrosis [24, 25]. Myocardial T2 is directly proportional to myocardial water content [26] and K^trans^ measures capillary permeability [27]; both are applied as measures of tissue inflammation.

Using these techniques, we show that stable EGPA is associated with myocardial fibrosis (elevated ECV and T1) and an absence of myocardial inflammation (normal T2 and K^trans^), findings which are in keeping with the known histology, described earlier. Our findings also show that the evident myocardial fibrosis comprises both diffuse interstitial and replacement components, and that it is myocardial fibrosis that drives the borderline left ventricular hypertrophy i.e. rather than cellular hypertrophy.

Greulich et al. reported elevated myocardial ECV and T1 in EGPA, although myocardial T2 was also elevated, indicating the presence of myocardial oedema [10]. ECV and T1 can be affected by oedema, thus it is unclear whether the elevated ECV and T1 that Greulich et al. report represented myocardial oedema or fibrosis. In a study by Cereda et al., elevated ECV was confined to patients with reduced LV ejection fraction [11]. The prevalence of non-ischaemic focal replacement fibrosis in our study (77%) is in keeping with that in previous studies [11, 28–30]. Although quantitative T2 mapping has only been applied in one previous study in EPGA, studies using semi-quantitative T2-weighted imaging, and positron emission tomography, have generally shown a low prevalence of inflammation in stable EGPA, which is in keeping with the described histology and our findings [5, 9, 29–31].

Myocardial fibrosis is strongly associated with adverse outcome across a range of cardiovascular conditions [32, 33] and further work is required to determine the association between myocardial fibrosis and outcome in EGPA. Arrhythmia is a common feature of EGPA and it is of note that we demonstrate an association between myocardial fibrosis and corrected QT interval.

The authors of pathological studies demonstrating that myocardial fibrosis develops rapidly during the course of EGPA have suggested immediate and aggressive immunosuppressant treatment may be beneficial in preventing the development of heart failure and other cardiac complications [2, 8]. Future studies applying MRI in the initial stages of EGPA are required in order to characterise the early myocardial manifestations and evaluate the impact of such an immunosuppressant strategy.

Importantly, one patient (8%) was found to have an otherwise unrecognised MI. MI that is not detectable from patient history or ECG is well described in non-EGPA populations. Cardiac MRI with LGE has a sensitivity such that it is able to detect 0.7 g of infarcted myocardium [34]. Several large community cohort studies (including people with or at risk of cardiovascular disease) utilising cardiac MRI have demonstrated a significant prevalence of otherwise unrecognised MI, and a high associated mortality risk [35]. Subendocardial LGE is well described in EGPA but studies rarely comment on its apparent aetiology (i.e. non-ischaemic eosinophilic subendocardial fibrosis vs. ischaemic), or its context (i.e. history of MI). It is well recognized that EGPA is associated with coronary artery disease, and while our study is small, it suggests that unrecognised MI may not be uncommon. Given that unrecognised MI is only detectable using cardiac MRI, and that its detection allows initiation of secondary preventative therapy, consideration should be given to performing cardiac MRI in all patients with EGPA.

Demonstrating the unique utility of cardiac MRI, the cardiac MRI findings were not evident from circulating biomarkers (e.g. high sensitivity troponin) or on ECG. Furthermore, despite there being evidence of significant myocardial injury and adaptation, ventricular size and ejection fraction were normal, which is in keeping with most previous studies in EGPA, demonstrating the low sensitivity of ventricular size and ejection fraction for detecting cardiac injury [10, 28, 30].

Our cardiopulmonary MRI protocol, which simultaneously interrogates cardiac and pulmonary inflammation, fibrosis and blood flow characteristics, provides a methodology to directly investigate cardiac and pulmonary pathophysiology, and their relationship. In the first study to apply pulmonary MRI in EGPA, we show that that in the setting of stable EGPA with effective immunosuppression, pulmonary tissue characterisation is normal with no evidence of inflammation or pulmonary fibrosis. Active pulmonary inflammation is associated with increased pulmonary T1, capillary permeability and pulmonary tissue blood flow [27, 36, 37]. Our findings, which showed no elevation in these markers, indicates stable EGPA is not associated with active pulmonary inflammation, which was in keeping with the clinical picture. Pulmonary T1 is reduced in the context of pulmonary tissue destruction, such as occurs with emphysema, and with pulmonary fibrosis [36, 38–42]. Histological studies have demonstrated that stable EGPA is not associated with emphysema or fibrosis [2, 6, 43, 44], which is in keeping with our findings that showed no reduction in pulmonary T1 and no increase in extracellular volume fraction in EGPA compared to controls. Indeed, a benefit of our combined cardio-pulmonary protocol is that it enabled us to demonstrate that myocardial fibrosis occurs independently of pulmonary fibrosis in EGPA.

We found a strong association between myocardial fibrosis and the severity of airflow limitation. This is a novel finding and the underlying mechanism is unclear. Associations between FEV_1_ and incident heart failure, and between FEV_1_ and adverse outcome in patients with HF, particularly heart failure with preserved ejection fraction, are widely recognised [45–49]. The mechanisms underlying these associations are also not known, but, in the context of chronic obstructive pulmonary disease (COPD), circulating inflammatory markers are associated with incident heart failure, myocardial injury (circulating troponin level) and airflow obstruction [50–52]. In COPD, it has been hypothesised that systemic inflammation leading to myocardial injury and subsequent myocardial fibrosis may be the unifying mechanism [53]. It is possible that a similar mechanism may be responsible for the relationship between myocardial fibrosis and airflow limitation that we have demonstrated in EGPA, indeed our findings may shed light on these wider pulmonary-cardiac associations, but further work is required to investigate these mechanisms.

## Limitations

The number of patients included was small, although we were able to demonstrate significant results despite the small numbers. EGPA is a rare condition with estimated prevalence of 2 to 22 per/million and the size of our study is in keeping with other recent studies [2, 10, 11]. It was not possible to obtain histological data; it was not felt ethically acceptable to conduct lung and endomyocardial biopsies as part of this study, and these procedures are not generally performed in stable EGPA patients.

## Conclusions

In this exploratory study that utilised a combined multiparametric cardiopulmonary MRI protocol, we demonstrate that stable EGPA is associated with focal replacement and diffuse interstitial myocardial fibrosis, but no evidence of myocardial or pulmonary inflammation or pulmonary fibrosis. Myocardial fibrosis is strongly related to airway obstruction and abnormal cardiac repolarisation. Further investigation is required to determine the mechanisms underlying the association between heart and lung disease in EGPA, the impact myocardial fibrosis has on prognosis, and whether an immediate immunosuppressive strategy could prevent myocardial fibrosis formation.

## Data Availability

The datasets generated and/or analysed during the current study are available from the corresponding author on reasonable request.
